# Do terrestrial animals avoid areas close to turbines in functioning wind farms in agricultural landscapes?

**DOI:** 10.1007/s10661-017-6018-z

**Published:** 2017-06-19

**Authors:** Rafał Łopucki, Daniel Klich, Sylwia Gielarek

**Affiliations:** 1 Center for Interdisciplinary Research, The John Paul II Catholic University of Lublin, Konstantynów 1J, 20-708 Lublin, Poland; 2 Department of Genetics and Animal Breeding, Warsaw University of Life Sciences—SGGW, Ciszewskiego 8, 02-786 Warsaw, Poland; 3 The Regional Directorates for Environmental Protection in Lublin, Bazylianówka 46, 20-144 Lublin, Poland

**Keywords:** Wind energy, Environmental impact assessment, European roe deer, European hare, Red fox, Common pheasant

## Abstract

Most studies on the effects of wind energy on animals have focused on avian and bat activity, habitat use, and mortality, whereas very few have been published on terrestrial, non-volant wildlife. In this paper, we studied the utilization of functioning wind farm areas by four terrestrial animals common to agricultural landscapes: European roe deer, European hare, red fox, and the common pheasant. Firstly, we expected that the studied animals do not avoid areas close to turbines and utilize the whole area of functioning wind farms with a frequency similar to the control areas. Secondly, we expected that there is no relation between the turbine proximity and the number of tracks of these animals. The study was conducted over two winter seasons using the snow-tracking method along 100 m linear transects. In total, 583 transects were recorded. Wind farm operations may affect terrestrial animals both in wind farm interiors and in a 700-m buffer zone around the edge of turbines. The reactions of animals were species specific. Herbivorous mammals (roe deer and European hare) avoided wind farm interiors and proximity to turbines. The common pheasant showed a positive reaction to wind turbine proximity. The red fox had the most neutral response to wind turbines. Although this species visited wind farm interiors less often than the control area, there was no relation between fox track density and turbine proximity. Greater weight should be given to the effects of wind farms on non-flying wildlife than at present. Investors and regulatory authorities should always consider the likely impacts of wind farms during environmental impact assessments and try to reduce these negative effects.

## Introduction

Wind power is becoming an increasingly important energy source in a growing number of countries (REN21 2014). The promotion of this renewable source for electricity production is a priority in the energy policy of many countries around the world (Karydis 2013; Mann and Teilmann 2013). For example, in the USA, wind energy development is targeted to meet 20% of energy demand by 2030 (Winder et al. 2014a, b), but some countries already meet high proportions of their energy needs with wind power, e.g., up to 20.9% in Spain and 33.2% in Denmark (REN21 2014).

Projected global growth in wind energy development has the potential to affect wildlife populations negatively. Most studies on the effects of wind energy have focused on avian and bat activity, habitat use and mortality (Kunz et al. 2007; Pearce-Higgins et al. 2012), whereas very little has been published on terrestrial, non-volant wildlife (Lovich and Ennen 2013). Terrestrial animals can be affected by wind power development in various ways: destruction and modification of habitat (habitat fragmentation and barriers to gene flow), noise effects, visual impacts, vibration and shadow flicker effects, electromagnetic field generation, macro- and micro-climate change, predator attraction, increase of fire risk, and increase of direct mortality on wind farm roads (de Lucas et al. 2005; Santos et al. 2010; Lovich and Ennen 2013). The amount of negative effects varies with wind energy projects depending on the type and size of the installation, location (whether it is situated in degraded or undisturbed habitat) and the life cycle stage of the installation (e.g., construction, operation, maintenance or decommissioning) (Helldin et al. 2012; Lovich and Ennen 2013).

Helldin et al. (2012) argued that wind farms affect large terrestrial mammals mainly through an increase in human activity within the wind farm area. During the construction phase, large-mammal carnivores and ungulates may temporarily avoid wind farms, but when construction and human presence cease, these animals acclimate to the wind energy infrastructure. Consequently, low-level impacts of wind farms during the operational phase were observed on the home ranges of large mammals and their behavior and nutritional ecology (Helldin et al. 2012; Walter et al. 2006). Previous studies have found both a significant effect of wind farms (Agha et al. 2015; Lovich et al. 2011; Rabin et al. 2006) and no effects on medium-sized and small animals (de Lucas et al. 2005; Ennen et al. 2012; Łopucki and Mróz 2016; Winder et al. 2014a, b). A limited number of studies conducted on a narrow group of species and species-specific differences in results do not allow reliable generalizations about the effects of wind turbines on terrestrial animals. Much more research is needed to assess fully the direct and indirect impacts of wind energy on terrestrial wildlife across various habitats and climatic regions.

The aim of this work was to present empirical data on the utilization of functioning wind farm areas by selected large- and medium-sized terrestrial animals living in agricultural landscapes in central Europe. The species analyzed in this study were the European roe deer *Capreolus capreolus* (Linnaeus, 1758), the European hare *Lepus europaeus* (Pallas, 1778) and red fox *Vulpes vulpes* (Linnaeus, 1758), and a heavy-bodied ground-feeding bird, the common pheasant *colchicus* (Linnaeus, 1758). We decided to study a group of species that exhibit different ecology and food preferences because we expected that reactions to wind turbines (avoidance or preference of wind turbine proximity, or no effect) might be species specific, depending on prey or predator position in the food chain or predation risk. Some authors suggest that wind energy development affects local predator communities, resulting in an indirect effect of decreased predation risk for prey species (Agha et al. 2015; Winder et al. 2014a, b). The targeted species have home ranges large enough to show possible differences in their utilization of functioning wind farm areas. On the other hand, these species have home ranges small enough for a wind farm to be an essential part of the areas they inhabit and large enough for them to avoid or search for turbines. Specifically, the home ranges of the studied species are as follows: roe deer, from 57 to 88 ha (Morellet et al. 2013); European hare, from 10 to 40 ha (Kunst et al. 2001; Rühe and Hohmann 2004); red fox, from 300 to 600 ha (Goszczyński 1989); common pheasant, from 0.5 to 45 ha (Okarma et al. 2008).

We conducted the study during the operational phase of wind farms and, in accordance with published studies (Helldin et al. 2012; Lovich and Ennen 2013; Łopucki and Mróz 2016; Walter et al. 2006; Winder et al. 2014a, b), we expected that the studied animals would not avoid areas close to turbines, would utilize whole area of the functioning wind farms with a frequency similar to the control areas, and that there would be no relation between wind turbine proximity and the number of tracks of these animals. We tested the null hypothesis of no effect (negative or positive) of wind turbines on the studied species. The study was conducted during winter, which is characterized by the lowest human presence within wind farm areas (no agricultural work), so that the operation of wind turbines can be considered a major factor affecting wildlife in this agricultural landscape. Moreover, winter is the most critical time of year for animals in this continental climate region, where significant disturbances in habitat utilization could result in a decrease in survival rates.

## Methods

### Study area

The study was conducted in southeastern Poland (central Europe) within the foothills of the Outer Western Carpathian Mountains (310–580 m above sea level) at three wind farms: “Łęki Dukielskie” (N 49° 36′ 52″, E21° 40′ 54″), “Rymanów” (N 49° 36′ 19″, E 21° 50′ 37″), and “Bukowsko” (N 49° 30′ 31″, E 22° 5′ 17″). The studied wind farms and their surroundings have already been described by Łopucki and Mróz (2016). Briefly, all of the farms consist of Repower MM92 wind turbines with a tower height of 100 m, a rotor diameter of 92.5 m, and single-turbine capacities of 2.05 MW. The “Łęki Dukielskie” wind farm consists of five wind turbines located at a distance of 18 to 600 m from the periphery of the forest. From the south, the turbines adjoin agricultural areas. The “Rymanów” wind farm consists of 13 wind turbines located in arable fields and meadows with small groups of shrubs. The “Bukowsko” wind farm consists of nine wind turbines located on two adjacent hills between forest and open agricultural areas.

The region of the study is characterized by variable agricultural landscapes that are not dominated by monoculture, with the following percentages of wooded areas (presented for the administrative districts (communes) where the wind farms are located): Dukla commune 36%, Rymanów 34%, Bukowsko 52%. The region has a continental climate of hot summers (mean 17.7 °C in July), cold winters (mean −5.8 °C in January) and a mean annual temperature of 7.0 °C. The mean annual precipitation is 697 mm and there is snow cover on average 90 days a year.

### Snow tracking

**Fig. 1:**
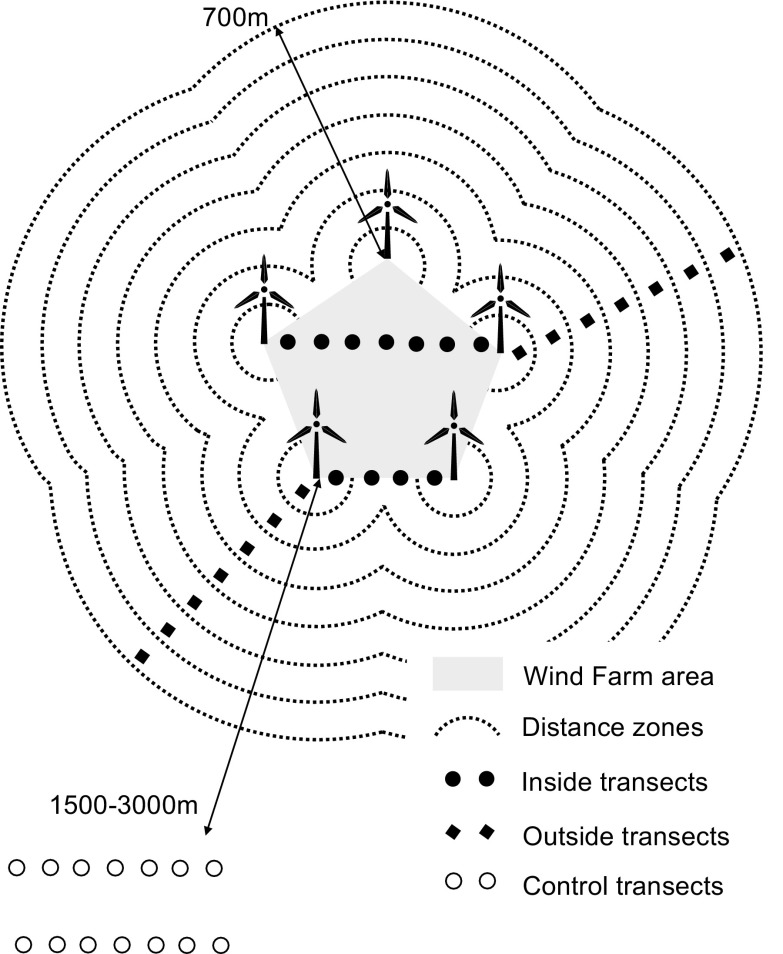
Schematic arrangement of the three groups of transects on each studied wind farm area: within-farm, outside-farm, and control transects (for description of transects, see text)

The main method used to estimate how animals utilize these areas was snow tracking along 100 m linear transects. These transects were categorized into three location types (Fig. 1, Table 1):

1. The first group of transects, referred to as “within-farm transects”, was among turbines inside wind farm areas regardless of turbine proximity. The farm area units were defined by minimal convex polygons (MCP) connecting the outermost turbines on each farm. In total, 208 transects in this type of location were recorded.
2. The second type of transect, referred to as “outside-farm transects”, was on the outskirts of the farms along a distance gradient directed from the edge of each farm (edge of turbines) to the outer area, up to 700 m. Common practice is to keep wind farms at least 700 m away from human settlements as this is regarded as a safe distance with no essential impact of the wind farm (Mroczek et al. 2012). In total, 255 transects in this type of location were recorded.
3. The third type of transect, referred to as “control transects,” were located in control areas 1.5–3 km away from each wind farm. Control transects were chosen in such a way that the type of vegetation, topography, altitude and the nature of the surroundings were as similar as possible to those around the wind turbines. In total, 120 transects in this type of location were recorded.

**Table 1:** The number of transects categorized into three location types (inside, outside, and control transects) surveyed on study wind farms over two winters (2014 and 2015)

	Wind farms			Total
	Łęki Dukielskie	Rymanów	Bukowsko	
No. of wind turbines covered in the study	5	13	5	23
No. of inside transects	56	142	10	208
No. of outside transects	58	124	73	255
No. of control transects	40	50	30	120
Total no of transects	154	316	113	583

Each transect was traversed only once. Transects were randomly oriented and located in each location type or distance zone. Such an arrangement ensures efficiency and accuracy when estimating the area utilization of terrestrial animals under hunting pressure (Fragoso et al. 2016). Snow tracking was conducted by three observers during two winter periods: 2013/2014 and 2014/2015. Animal tracks were counted on the second day after snowfall, when the air temperature was below but close to 0 °C (when snow tracks are the most readable and permanent) with no high wind velocity (to prevent tracks being covered with snow), but with sufficient wind velocity for turbines to turn. During both winter periods, days with similar snow pattern, temperature, and wind velocity were chosen in order to avoid differences in animal activity. The position of every encountered track (on the transect line) was recorded with a GPS receiver and the species was identified. Tracks left by the studied animals differ from each other and are relatively simple to identify properly. When there was any doubt about the species, the track was followed to where it could be properly identified. If necessary, the track length, width, and step length were measured.

### Data analysis

The basic unit of data used in the analysis was the number of tracks found within each 100 m transect. Analyses were performed for each study species separately.

The influence of wind farms on the targeted species was assessed in two ways: (a) the impact of wind farms as a landscape element and (b) the impact of solitary turbines along a distance gradient.

- The analysis of the impact of wind farms as landscape elements on animals was based on data from within-farm transects and data from control area transects. In each distance zone, the number of tracks from transects within the zone was compared. For each species, we used a generalized linear model to compare the mean track density of a given species on within-farm transects and control area transects. The number of tracks per transect was a response variable, and location (farm/control) was a fixed factor. We used a negative binomial distribution with log link after comparison with the Poisson distribution model using the Akaike information criterion (AIC) and the Bayesian information criterion (BIC). Both distributions are used in count data, but negative binomial models can also cope with overdispersion in the count data (Zuur et al. 2009) and showed lower values of both information criteria (AIC and BIC) for each species.
- The impact of solitary turbines on animals was assessed using a shapefile layer related to seven 100 m distance zones from turbines (distance gradient). The layers were created in ArcGis using a Multiple Ring Buffer Analysis. Shapefile layers of recorded tracks and transects were created using DNR GPS and compared to shapefiles of distance zones. Also due to the data type, we used a generalized linear model in which the number of tracks per transect was a response variable and distance was a set as a covariate. We used a negative binomial distribution with log link, which fit much better than the model with Poisson distribution based on the Akaike information criterion (AIC) and the Bayesian information criterion (BIC).

All statistical tests were performed using the SPSS statistical software package.

## Results

In total, across all 583 transects, 4347 tracks from 15 animal species were identified, with the majority (89.5%) of tracks belonging to the targeted species. There were 2062 tracks for the European roe deer (47.4%), 1062 tracks for the European hare (24.4%), 583 tracks for the red fox (13.4%), 185 tracks for the common pheasant (4.3%), and 455 tracks for all other species. The remaining tracks identified in the studied transects belonged to the following species: wild boar, red deer, wolf, raccoon dog, marten, squirrel, weasel, gray partridge, domestic dog, domestic cat, and undetermined small mammals. The tracks of these species were not sufficiently numerous for analysis.

### Impact of wind farms as landscape elements

**Fig. 2:**
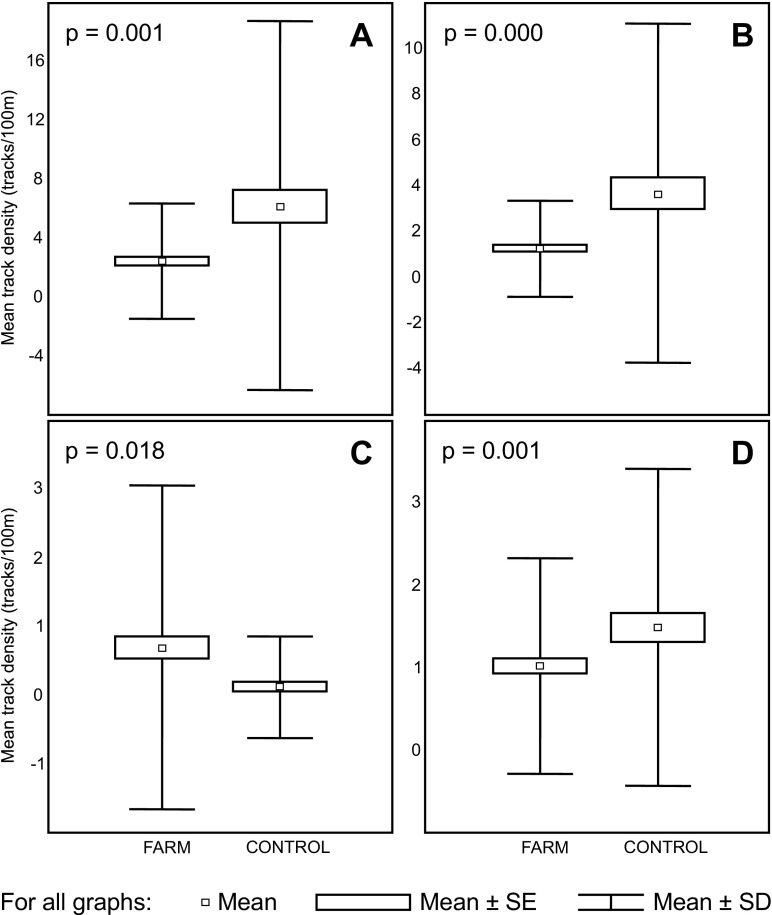
Mean track density (tracks/100 m) for roe deer (**a**), European hare (**b**), common pheasant (**c**), and red fox (**d**) within wind farm areas (FARM) and within control areas (CONTROL), and pairwise comparisons in GLMs with Bonferroni adjustment (*p* values shown on the graph, *N* = 208 for wind farm area and *N* = 120 for control area in all cases)

The wind farms influenced the presence of all the targeted species. Within the wind farms, the mean track density per 100 m of roe deer was 2.47, which was less than half of that in areas without the impact of a wind farm (6.21 tracks/100 m). This difference was statistically significant (*p* = 0.001) despite a high standard deviation of this parameter within the control area (SD = 12.54) (Fig. 2a). The European hare also showed lower usage of wind farm areas in comparison to control areas (1.27 and 3.7 tracks/100 m, respectively), with a high statistical significance (*p* = 0.000) (Fig. 2b). The impact of the wind farms on common pheasants was also observed (*p* = 0.018), but the mean track numbers were higher within wind farm areas than in control areas (0.69 and 0.12 tracks/100 m, respectively) (Fig. 2c). The track density of the red fox within farm areas was 1.02 and was significantly lower than in the control areas (1.49 tracks/100 m, *p* = 0.001) (Fig. 2d).

### Impact of solitary turbines on distance gradients

**Fig. 3:**
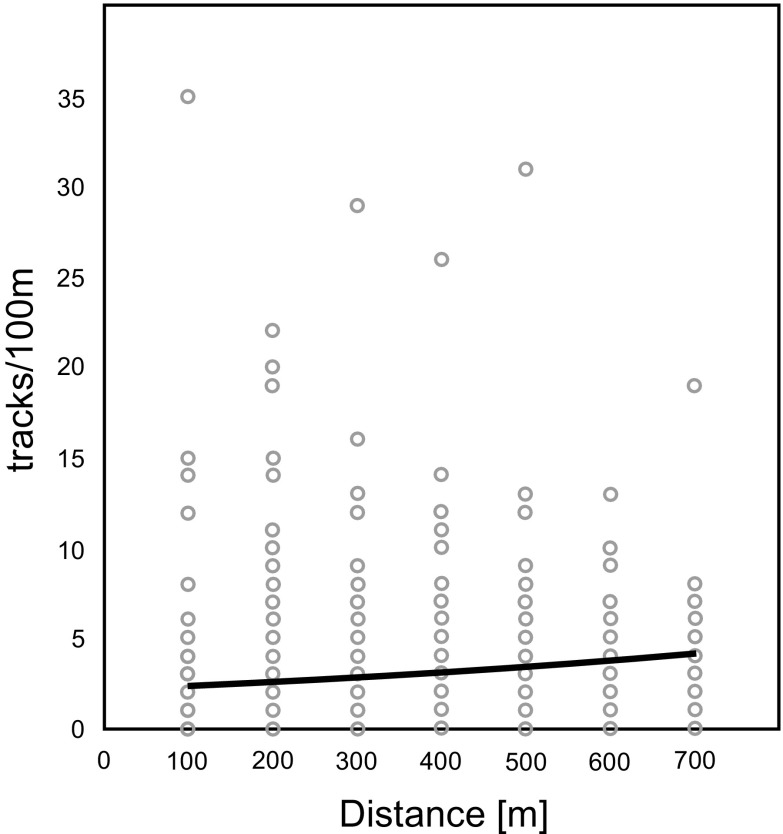
Effect of distance from turbines on track numbers of roe deer on 100 m transects (tracks/100 m), the *curve* shows the generalized linear model fit (likelihood ratio *χ2* = 9.245; *p* = 0.003; *N* = 480)

**Fig. 4:**
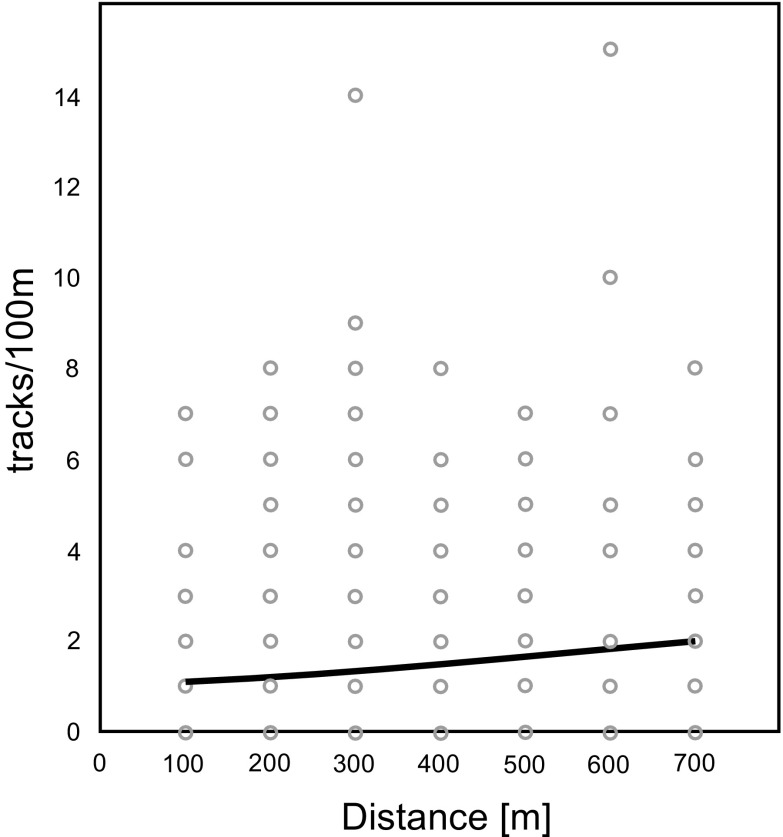
Effect of distance from turbines on track numbers of European hare on 100 m transects (tracks/100 m), the *curve* shows the generalized linear model fit (likelihood ratio *χ2* = 22.184; *p* = 0.000; *N* = 480)

Roe deer showed a significant increase in track density farther away from wind turbines (*p* = 0.003) (Fig. 3). Expected track density in the model increased from c.a. 2.5 tracks/100 m close to the turbines (0–100 m zone) to over 4 tracks/100 m in the most distant zone (600–700 m). Similar avoidance of turbines was clear for the European hare, for which track density was also positively correlated with increasing distance from the turbine (*p* = 0.000) (Fig. 4). The lowest track density was expected in the model for European hares within the first 100 m from turbines (1.1 tracks/100 m), with a gradual increase in more distant zones (500–700 m) to a track density of over 2 tracks/100 m.

**Fig. 5:**
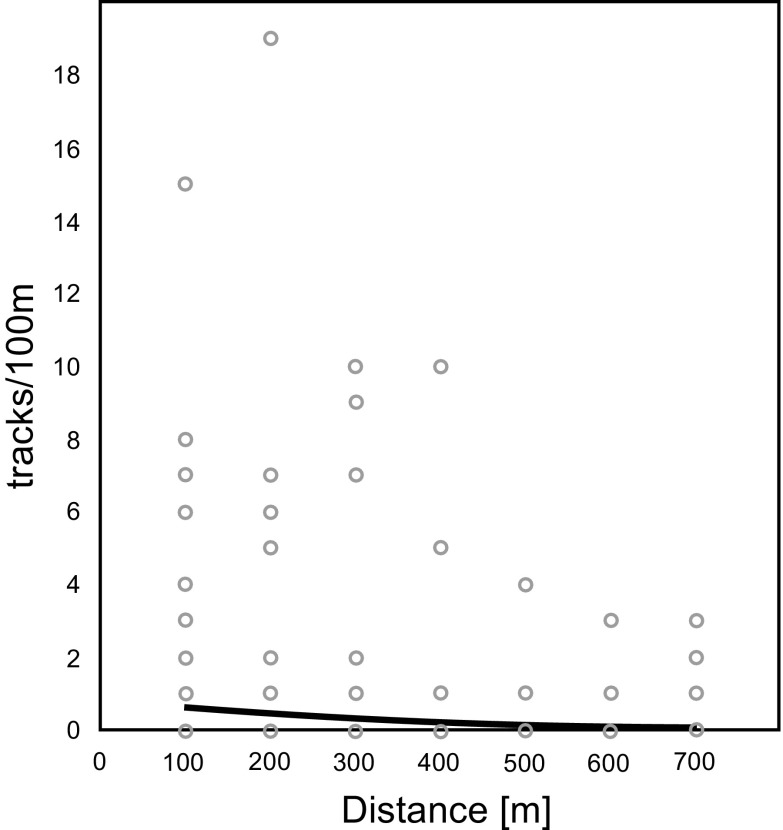
Effect of distance from turbines on track numbers of common pheasant on 100 m transects (tracks/100 m), the *curve* shows the generalized linear model fit (likelihood ratio *χ2* = 182.367; *p* = 0.000; *N* = 480)

Common pheasant track numbers per 100 m decreased significantly with increasing distance from turbines, (*p* = 0.000) (Fig. 5). A gradual decrease of expected track density was observed from the first 100 m (0.67 tracks/100 m) to a distance of 700 m (0.06 tracks/100 m).

**Fig. 6:**
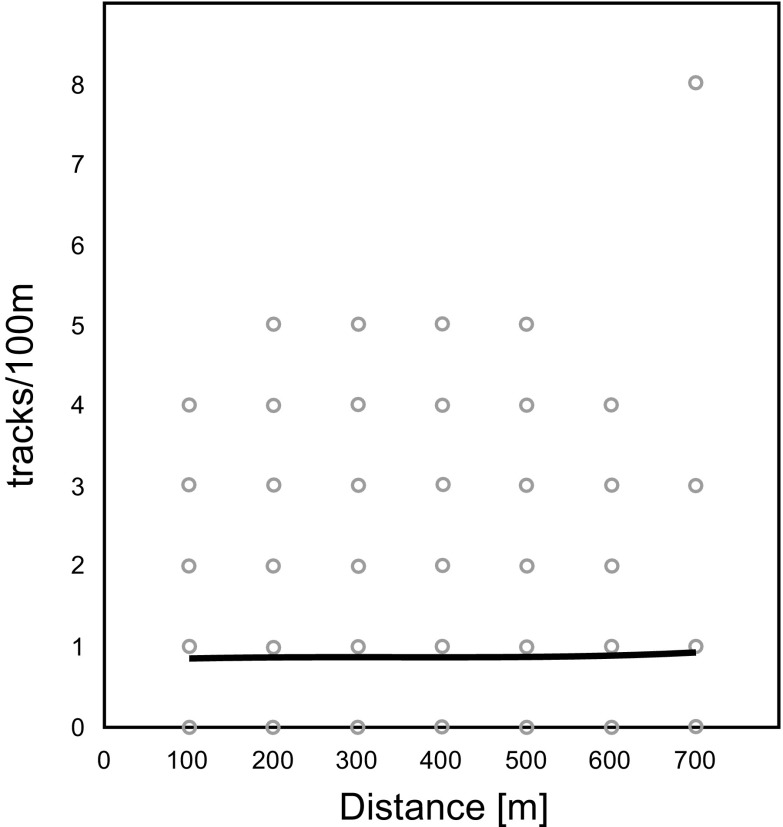
Effect of distance from turbines on track numbers of red fox on 100 m transects (tracks/100 m), the *curve* shows the generalized linear model fit (likelihood ratio *χ2* = 0.129; *p* = 0.72; *N* = 480)

No relationship was found between track density of red foxes and distance gradients to turbines (*p* = 0.72) (Fig. 6). Expected track density in the model oscillated in all zones between 0.85 and 0.92 tracks/100 m.

## Discussion

Terrestrial animals can potentially be affected by wind power development in various ways (Lovich and Ennen 2013), but the empirical evidence suggests a lack of such effects during the operational phase of wind farms or a swift habituation of animals to the disturbance, thus suggesting a limited impact (Helldin et al. 2012; Lovich and Ennen 2013; Łopucki and Mróz 2016; Menzel and Pohlmeyer 1999; Walter et al. 2006; Winder et al. 2014a, b). For this reason, we would expect no effect (neither negative nor positive) of wind turbines on the studied species. However, our study showed that wind farms can significantly alter habitat use of terrestrial animals not only during the construction phase (Helldin et al. 2012), but also during the operational phase.

In our study, terrestrial animals did indeed use the wind farm areas (tracks of all analyzed species were found close to turbines), which is in accordance with the published literature regarding the different animal species (Helldin et al. 2012; Lovich and Ennen 2013; Rabin et al. 2006; Winder et al. 2014a, b), but the frequency of use of areas near wind turbines was different than the use of land far away from turbines. Our results show that wind farm operations affect terrestrial animals both in wind farm interiors (among the turbines), as well as within at least a 700-m buffer zone around each wind farm. The reaction of the studied animals to wind farms, however, is specific and depends on the species.

### Herbivorous mammals

Wind farm interiors were less intensively frequented by roe deer and European hare than control areas. Both species also showed a clear linear decrease of track density with decreasing distance to the turbine. Acoustic factors seem to be the most probable reason for the lower presence of these species in the wind farm areas and with decreasing distance to turbines. Permanent and high noise levels may be bothersome enough for these species to visit less frequently and spend less time there (resulting in lower track density) because the noise may cause metabolic stress and be harmful for animals (Du et al. 2010; Kight and Swaddle 2011). However, it is more likely that herbivorous mammals have difficulty perceiving sounds in noisy environments. Human-related noise may restrict the habitat use and movement of species and represent a life-threatening hazard in predator-prey interactions, particularly regarding herbivorous mammals (Barber et al. 2010; Chan et al. 2010; Clevenger and Waltho 2005; Rabin et al. 2006). Both the roe deer and the European hare rely more on hearing than other senses, particularly to avoid danger (Molinari-Jobin et al. 2004) and, thus, are susceptible to such a noise effect. Both are also under high predatory pressure in the Carpathian region (Bombik et al. 2014; Kauhala and Kowalczyk 2011). Herbivorous mammals generally increase their vigilance in environments that are under a high predatory pressure (Laundré et al. 2001; Wolff and Horn 2003). Avoidance of risky habitats is an important part of their antipredatory strategy (Aulak and Babińska-Werka 1990; Bongi et al. 2008; Klich and Grudzień 2013) that may lead to some areas being depleted of prey due to vigilance rather than predation (Brown et al. 1999). For roe deer and the European hare, the proximity of turbines may thus represent a risky habitat due to the animals’ impaired ability to hear approaching predators. This phenomenon may be more likely during hours of darkness or low light, when both species are most active during winter (Chapman and Flux 2008; Pépin and Cargnelutti 1994; Wallach et al. 2010). Previous studies (Bergen 2002; Menzel and Pohlmeyer 1999) showed no significant difference in roe deer distribution or habitat use in areas with wind farms compared to reference areas without wind power. However, neither roe deer nor the European hare were under high predation pressure that could displace them to other habitats (Keuling et al. 2011; Ludwig et al. 2009).

### Common pheasant

The common pheasant showed the opposite reaction to wind farms compared to roe deer and the European hare. Wind farm interior areas were visited more often by these birds than control areas and a positive relationship between pheasants and individual turbines was found (outside-farm transect results). Such results suggest that there is no impact of turbine noise on this species or that the impact is mitigated by other factors. We hypothesize that there are probably two primary factors. Firstly, a positive impact may result from the fact that wind farms may reduce the presence of birds of prey both through direct mortality due to collisions and because they avoid foraging near wind turbines (Drewitt and Langston 2006; Garvin et al. 2011; Kuvlesky et al. 2007; Lovich 2015; Osborn et al. 1998; Pearce-Higgins et al. 2012; Smallwood 2007, 2013; Smallwood et al. 2009). Consequently, animals living close to turbines may be under less pressure from avian predators (Agha et al. 2015; de Lucas et al. 2005; Winder et al. 2014a, b). Secondly, we hypothesize that the availability of grit near turbines may be the most important attractant for pheasants, especially in winter. During our study, we often observed that although the entire wind farms were covered by snow, the areas at the base of turbines had sparse snow cover. The areas around these turbine bases were also abundant in various sizes of grit, including small particles suitable for pheasants (the area around each turbine base is hardened with macadam or breakstone). Pheasants ingest stones more often and in greater amounts than many other wild bird species (Gionfriddo and Best 1999; Hussain and Sultana 2013). We speculate that pheasants rarely but regularly visit turbine bases to ingest gastroliths, which are much easier to find than in other parts of wind farms. Such regular visiting of habitats abundant in grits has been demonstrated in pheasants and other bird species (Best and Gionfriddo 1991).

### Red fox

The red fox visited interior wind farm areas significantly less often than control areas. Prey availability is probably the main reason for the presence of red foxes due to the influence of prey distribution on predator activity (Theuerkauf et al. 2003). The mean track density of the hare (the fox’s most important prey species (Goszczyński 1989)) was lower within the farm areas than in control areas. On the other hand, it is known that red foxes hunt a wide range of prey and can switch diets, for example to groups of small mammals (Dell'Arte et al. 2007; Jędrzejewski and Jędrzejewska 1992). Previous findings suggest no influence of wind farms on the diversity and abundance of small mammal species (Łopucki and Mróz 2016) in comparison to other types of human impacts (Łopucki et al. 2013; Łopucki and Kiersztyn 2015; Łopucki and Kitowski 2017). Nevertheless, red foxes rely mainly on hearing to hunt of this type of prey in the winter season (Malkemper et al. 2015); therefore, hindered hearing may lower the chances of hunting success. In our opinion, both factors (lower track density of the European hare and lower success in preying on small mammals) lead to lower track density of this species within farm areas. This raises the question of why there was no relationship between track density and distance to turbines (as shown for roe deer and the European hare) if the noise effect influences the area utilization of the red fox. The red fox, as a medium-sized predator, uses the habitat in a different way than herbivores and actively travels over long distances looking for prey (Goszczyński 1989; Servín et al. 1991). When searching for food, foxes tend to use linear landscape elements such as roads that allow them to cover longer distances, particularly in less accessible areas (Frey and Conover 2006; Halpin and Bissonette 1988). We speculate that foxes prefer wind farm roads to snow-covered fields when moving through wind farm areas. Wind farm roads lead directly to the turbines; therefore, the numbers of tracks in the various distance zones are similar. Another explanation is the fact that foxes intentionally move towards wind turbines to look for carrion (remains of birds killed by wind turbine blades). During our study, we never observed any bird remains near turbines; therefore, we believe that this source of food has little effect on red foxes in winter.

## Conclusions

Reactions of terrestrial animals to wind farms are diverse and species dependent. Foraging methods and environmental factors (e.g., predator presence) may significantly affect the behavior of animals. In this study, among the studied vertebrates within wind farms, it is possible to identify species negatively affected by the presence of turbines (wind farm interior or a single turbine) and species for which the presence of turbines seems to be beneficial or neutral. Herbivorous mammals (roe deer and European hare) less frequently visited wind farm areas and areas close to wind turbines, probably due to both the physiological effects of excessive noise and their impaired ability to hear approaching predators. The common pheasant is actively attracted to wind turbines, probably because of lower pressure from avian predators and readily available sources of gastroliths near turbine bases. The red fox showed the most neutral response to wind turbines. Though this species visited wind farm interiors less often than control areas (probably as a result of lower prey availability), there was no relation between fox track density and proximity of turbines.

Further similar studies at different stages of wind farm development and use are needed because the impact on terrestrial mammals of wind turbines in newer farms (the studied farms have been in operation no longer than 5 years) may be more distinct because animals have not yet adapted to this novel anthropogenic element in the environment. More data from different study sites with different predatory pressures is also needed to understand fully the effects of wind power on terrestrial animals.

This study shows that greater weight should be given to the effects of wind farms on non-volant wildlife than is currently the case. Investors and regulatory authorities should always consider and attempt to mitigate the likely impact of wind farms on terrestrial animals during environmental impact assessments. The impact of a wind farm should be considered in terms of not only the construction but also the operational phase, at least for the first 5 years after construction.
